# A stable phylogenomic classification of Travunioidea (Arachnida, Opiliones, Laniatores) based on sequence capture of ultraconserved elements

**DOI:** 10.3897/zookeys.760.24937

**Published:** 2018-05-28

**Authors:** Shahan Derkarabetian, James Starrett, Nobuo Tsurusaki, Darrell Ubick, Stephanie Castillo, Marshal Hedin

**Affiliations:** 1 Department of Biology, San Diego State University, San Diego, California 92182-4614, USA; 2 Department of Biology, University of California, Riverside, Riverside, California 92521, USA; 3 Department of Biological Sciences, Auburn University, Auburn, Alabama 36849, USA; 4 Laboratory of Zoological Systematics, Faculty of Agriculture, in Faculty of Regional Sciences Building, Tottori University, Tottori, 680-8551, Japan; 5 Department of Entomology, California Academy of Sciences, San Francisco, California 94118, USA; 6 Department of Entomology, University of California, Riverside, Riverside, California 92521, USA; 7 Present address: Department of Organismic and Evolutionary Biology, Museum of Comparative Zoology, Harvard University, 26 Oxford Street, Cambridge, MA 02138, USA

**Keywords:** cave evolution, harvestmen, historical biogeography, Holarctic, target enrichment, taxonomy

## Abstract

Molecular phylogenetics has transitioned into the phylogenomic era, with data derived from next-generation sequencing technologies allowing unprecedented phylogenetic resolution in all animal groups, including understudied invertebrate taxa. Within the most diverse harvestmen suborder, Laniatores, most relationships at all taxonomic levels have yet to be explored from a phylogenomics perspective. Travunioidea is an early-diverging lineage of laniatorean harvestmen with a Laurasian distribution, with species distributed in eastern Asia, eastern and western North America, and south-central Europe. This clade has had a challenging taxonomic history, but the current classification consists of ~77 species in three families, the Travuniidae, Paranonychidae, and Nippononychidae. Travunioidea classification has traditionally been based on structure of the tarsal claws of the hind legs. However, it is now clear that tarsal claw structure is a poor taxonomic character due to homoplasy at all taxonomic levels. Here, we utilize DNA sequences derived from capture of ultraconserved elements (UCEs) to reconstruct travunioid relationships. Data matrices consisting of 317–677 loci were used in maximum likelihood, Bayesian, and species tree analyses. Resulting phylogenies recover four consistent and highly supported clades; the phylogenetic position and taxonomic status of the enigmatic genus *Yuria* is less certain. Based on the resulting phylogenies, a revision of Travunioidea is proposed, now consisting of the Travuniidae, Cladonychiidae, Paranonychidae (Nippononychidae is synonymized), and the new family Cryptomastridae Derkarabetian & Hedin, **fam. n.**, diagnosed here. The phylogenetic utility and diagnostic features of the intestinal complex and male genitalia are discussed in light of phylogenomic results, and the inappropriateness of the tarsal claw in diagnosing higher-level taxa is further corroborated.

## Introduction

The arachnid order Opiliones is taxonomically rich, comprising 46 families, over 1,640 genera, and more than 6,600 described species (summarized in Kury 2000, Machado et al. 2007, Kury 2013). Within Opiliones, considerable phylogenetic progress has been made over the past ~10 years, summarized by Pinto-da-Rocha et al. (2007) and reviewed/updated in Giribet et al. (2010) and Giribet and Sharma (2015). This progress includes transcriptome-based phylogenomic approaches used in Hedin et al. (2012a), Sharma and Giribet (2014) and Fernández et al. (2017). Opiliones diversity falls into four primary clades, including the “mite harvestmen” (Cyphophthalmi), typical “daddy longlegs” (Eupnoi and Dyspnoi), and the “short-legged” or “armored” harvestmen (Laniatores). Laniatores is strongly supported as monophyletic, is the most species-rich group of harvestmen (with more than 4,100 described species) and can be found on all continents except for Antarctica. Many laniatoreans are tropical, where these animals are conspicuous and occupy a wide variety of habitats. Temperate laniatoreans are less noticeable, and in the Holarctic, are mostly small-bodied (~1.5–4 mm) predators restricted to cryophilic habitats (e.g., under decaying logs or rocks, in leaf litter, in caves, etc.).

**Figure 1.**
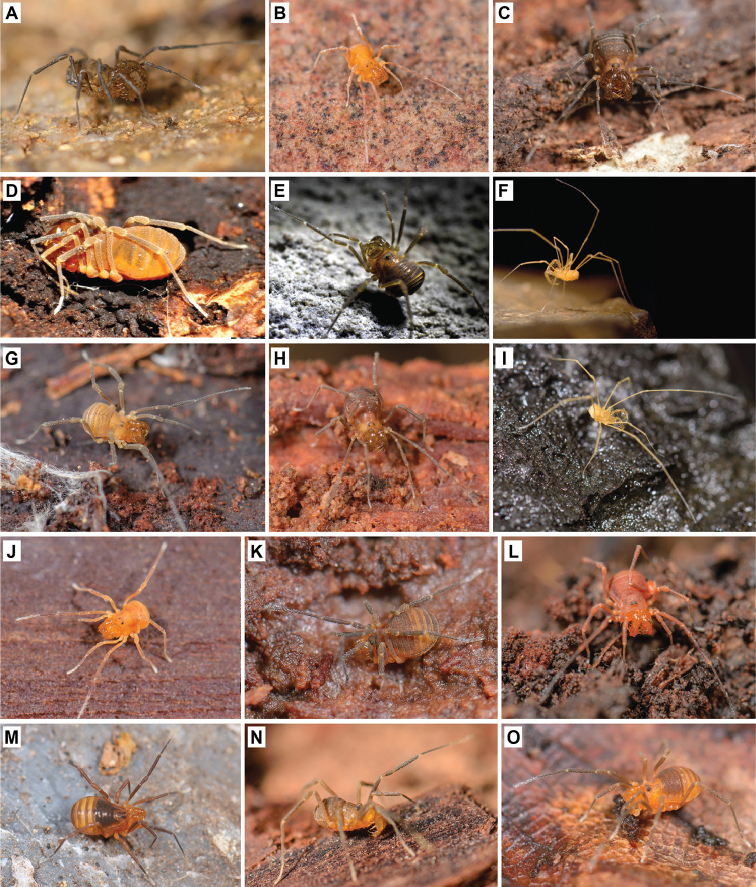
Photographs of live travunioid harvestmen. **A** *Theromaster brunneus* **B** *Erebomaster* sp. **C** *Cryptomaster leviathan* **D** *Holoscotolemon lessiniense* **E** *Peltonychia leprieurii* **F** *Trojanella serbica* **G** *Briggsus* sp. **H** *Isolachus spinosus* **I** *Speleonychia sengeri* **J** *Yuria pulcra* **K** *Paranonychus brunneus* **L** *Sclerobunus nondimorphicus* **M** *Metanippononychus* sp. **N** *Zuma acuta* **O** *Kainonychus akamai*. All photos by MH, except **D, E** (courtesy of and copyright A. Schönhofer), and **F** (courtesy of and copyright I. Karaman).

**Figure 2.**
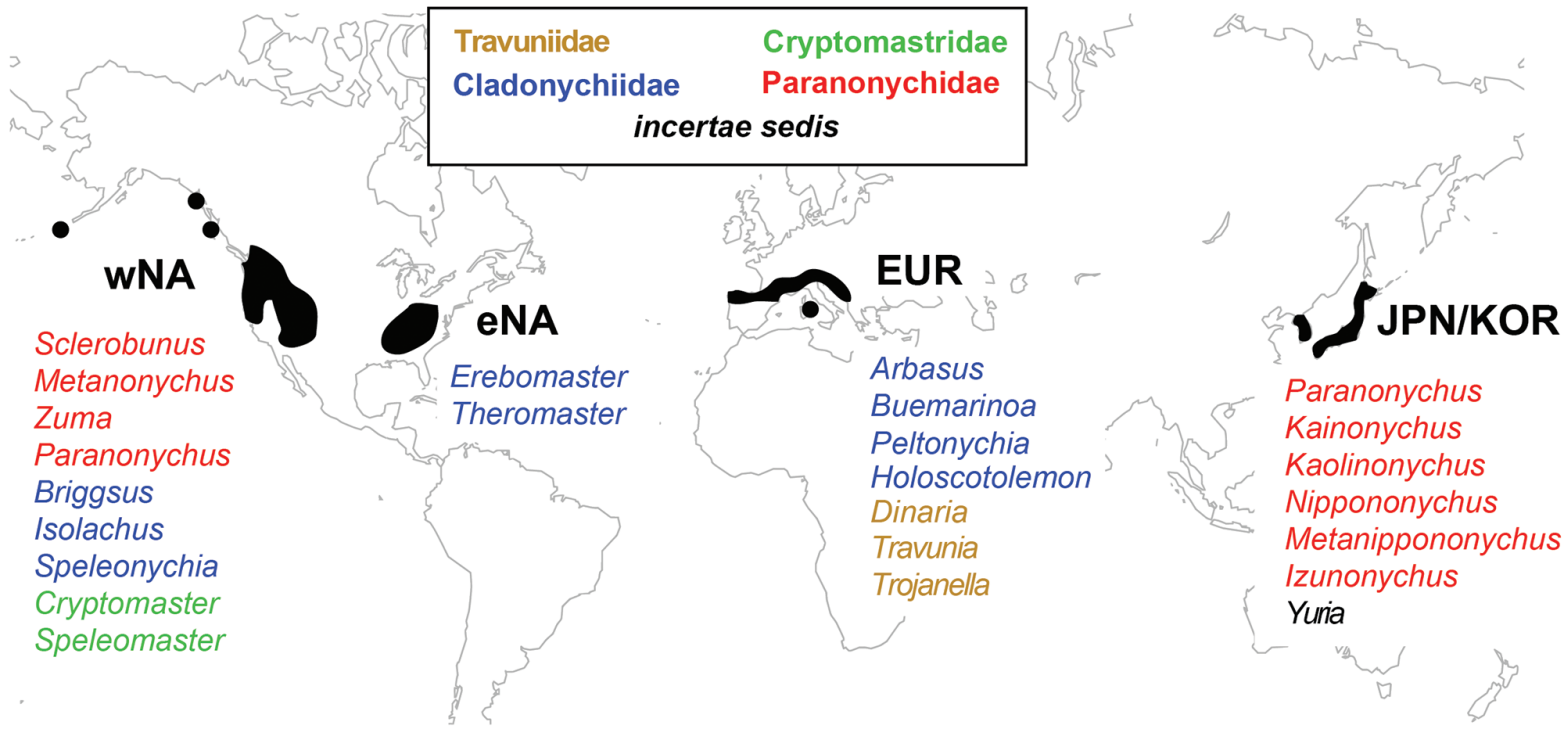
Geographic distribution of travunioid genera. Colors correspond to classification proposed in this study. Abbreviations: wNA = western North America, eNA = eastern North America, EUR = central and southern Europe, JPN/KOR = Japan and South Korea.

The molecular phylogenetic research of Giribet et al. (2010), which focused on relationships within Laniatores, formed the framework for further systematic research in these arachnids. Following this study, Sharma and Giribet (2011) conducted a phylogenetic analysis representing the most inclusive study of Laniatores to date. These authors recovered four primary laniatorean lineages (Fig. 1A), including the Synthetonychiidae Forster, 1954 (New Zealand), Triaenonychidae Sørensen, 1886 (mostly south temperate), Travunioidea Absolon & Kratochvíl, 1932 (north temperate), and Grassatores Kury, 2002 (broadly distributed, most diversity in the tropics). Although the sampling of travunioid taxa in these studies was incomplete (6-7 of 24 travunioid genera sampled), these molecular phylogenetic results and morphology (reviewed in Giribet and Kury 2007) support the monophyly of a north temperate lineage that constitutes the focal group of this study, the Travunioidea (Figs 1, 2).

Classification and generic level diagnoses within the Travunioidea have traditionally been based on structure of the tarsal claws of hind legs III and IV, particularly the number of side branches on the median prong. It is now widely-recognized that tarsal claw structure is a poor taxonomic character in this clade, as claw structure is highly homoplastic and variable at all taxonomic levels (e.g., Shear 1977, Maury 1988, Hunt and Hickman 1993, Karaman 2005, Shear and Derkarabetian 2008). For example, many travunioids were formerly grouped with the Triaenonychidae, sharing trident-shaped tarsal claw morphology. The transfer of all “north temperate triaenonychids” to the Travunioidea was first hinted at by Staręga (1971), proposed based on intestinal morphology by Dumitrescu (1975, 1976), and has since been supported with additional morphological (Giribet and Kury 2007, Mendes 2009) and molecular phylogenetic data (e.g., Derkarabetian et al. 2010, Giribet et al. 2010, Sharma and Giribet 2011). Other somatic morphological characters have been used to diagnose travunioid taxa (e.g., free lateral sclerites), but these characters may be retained plesiomorphic states (Kury 2007a) and/or potentially neotenic (Rambla 1980, Hunt and Hickman 1993). To illustrate the difficulty in the using these characters specifically in travunioids, Karaman (2005) described the monotypic genus *Trojanella* Karaman, 2005 yet left it unplaced within Travunioidea due to the uncertainty and homoplasy surrounding the diagnostic characters typically used to assign travunioid taxa.

Travunioidea has had a long and complicated taxonomic history dating back to 1861 with the description of the first species. Many European species were described by multiple authors throughout the late 1800s and early 1900s, resulting in many nomenclatural errors, comprehensively discussed in Kury and Mendes (2007). The vast majority of travunioid diversity was described during the mid-1960s to mid-1970s by Briggs (1969, 1971a, 1971b, 1974) in North America and Suzuki (1964, 1972, 1975a, 1975b, 1976) in Japan. Following this burst of taxonomic research very few studies focused on travunioids with the exception of two describing new European species (Tedeschi and Sciaky 1994, Karaman 2005), until sustained research began in the mid-2010s. During the mid-late 2010s, continued morphological work and the incorporation of genetic data confirmed the unsuitability of tarsal-claw taxonomy and resulted in several nomenclatural changes including new familial names (Giribet et al. 2010) and synonymies at the subfamilial, generic, and species levels (Shear and Derkarabetian 2008, Derkarabetian and Hedin 2014). Recent studies incorporating genetic and morphological analyses have led to the discovery, delimitation, and description of new travunioid species from western North America (Derkarabetian and Hedin 2014, Starrett et al. 2016).

Kury et al. (2014) recently provided a checklist of Travunioidea, made some taxonomic revisions, and provided a new taxonomy which serves as the starting point for this study. In this classification, Travunioidea includes 77 species/subspecies in 24 genera classified into three families, the Travuniidae Absolon & Kratochvíl, 1932 (including the historical Travuniidae, Cladonychiidae Hadži, 1935, and Briggsidae Özdikmen & Demir, 2008 as subfamilies), Paranonychidae Briggs, 1971, and Nippononychidae Suzuki, 1975. A summary of the historical classifications is presented in Table 1. While the monophyly of Travunioidea is almost certain, internal phylogenetic relationships remain largely unresolved. The most comprehensive phylogenetic analyses were conducted by Derkarabetian et al. (2010) and included samples from ten genera. Although this study was not focused on relationships among Travunioidea and only included samples from North America, the resulting phylogeny indicated a need for a taxonomic revision as multiple families and subfamilies were recovered as as para- or polyphyletic.

**Table 1.** Historical classification of the Travunioidea. Traditional classification refers to the taxonomy in place after the mid-1970s.

Traditional	Kury et al. (2014)
**Travunioidea**	**Travunioidea**
**Travuniidae**	**Travuniidae**
*Abasola*	**Travuniinae**
*Arbasus*	*Arbasus*
*Buemarinoa*	*Buemarinoa*
*Dinaria*	*Dinaria*
*Kratochviliola*	*Peltonychia*
*Peltonychia*	*Speleonychia*
*Travunia*	*Travunia*
*Speleonychia*	*Trojanella*
*Yuria*	**Cladonychiinae**
**Cladonychiidae**	*Cryptomaster*
*Cryptomaster*	*Erebomaster*
*Erebomaster*	*Holoscotolemon*
*Holoscotolemon*	*Speleomaster*
*Speleomaster*	*Theromaster*
*Theromaster*	**Briggsinae**
**Pentanychidae**	*Briggsus*
*Pentanychus*	*Isolachus*
*Isolachus*	
**Triaenonychoidea** (in part)	**Paranonychidae**
“**northern” Triaenonychidae**	**Sclerobuninae**
**Sclerobuninae**	*Sclerobunus*
*Sclerobunus*	*Zuma*
*Cyptobunus*	**Paranonychinae**
*Zuma*	*Paranonychus*
**Paranonychinae**	*Metanonychus*
*Paranonychus*	*Kaolinonychus*
*Metanonychus*	*Kainonychus*
*Kainonychus*	**Nippononychidae**
**Kaolinonychinae**	*Nippononychus*
*Kaolinonychus*	*Metanippononychus*
*Mutsunonychus*	*Izunonychus*
**Nippononychinae**	*Yuria*
*Nippononychus*	
*Metanippononychus*	
*Izunonychus*	

A robust genus-level phylogeny of Travunioidea and a stable classification would provide an important anchor for future taxonomic and evolutionary studies in this group. The stability of any phylogenetics-based classification relies upon high confidence and support for internal relationships. In other animal groups, genomic- or subgenomic-scale approaches have produced phylogenies with generally higher nodal support and have resolved difficult relationships (e.g., Blaimer et al. 2015, Garrison et al. 2016, Hamilton et al. 2016, Baca et al. 2017, Branstetter et al. 2017, Breinholt et al. 2017, Hedin et al. 2018). In this paper we utilize DNA sequences derived from capture of ultraconserved elements (UCEs; Faircloth 2017) to reconstruct phylogenomic relationships within Travunioidea. The phylogenetic utility of UCE data at multiple evolutionary scales has been demonstrated in other arachnid lineages (Starrett et al. 2017, Hedin et al. 2018). Our taxon sample includes 21 of 24 described genera and all currently and historically recognized travunioid families and subfamilies, plus outgroups. Although previous studies have examined relationships among Laniatores using a wider range of taxa (Giribet et al. 2009, Sharma and Giribet 2011), this study includes the most complete taxon set to date for Travunioidea.

## Materials and methods

### Taxon sampling

Fifty-seven specimens were included in this study (Suppl. material 1), including 40 Travunioidea, 14 non-travunioid Laniatores (eight Triaenonychidae, two Synthetonychiidae, and four Grassatores), and single representatives from each of the other three harvestmen suborders. Forty-nine samples were newly sequenced for this study; raw reads for other taxa are from Starrett et al. (2017). We included two samples for most travunioid genera (in most cases from two different species), except for *Trojanella* and *Travunia* Absolon, 1920, both of which were represented by a single specimen. We were unable to include three European travuniid genera (*Arbasus* Roewer, 1935, *Buemarinoa* Roewer, 1956, and *Dinaria* Roewer, 1935) as these are extremely rare and difficult to obtain cave-dwelling taxa.

### Molecular data collection

Genomic DNA was extracted from whole bodies using the Qiagen DNeasy Blood and Tissue Kit (Qiagen, Valencia, CA). For several larger specimens (body size greater than 3–4 mm) only legs, pedipalps, and chelicerae were used in extractions. Extractions were quantified using a Qubit Fluorometer (Life Technologies, Inc.) Broad Range kit, and quality was assessed via gel electrophoresis on a 0.8% agarose gel. Up to 500 ng of genomic DNA was used in sonication procedures, using a Bioruptor for 7 cycles at 30 seconds on and 90 seconds off, or a Covaris M220 Ultrasonicator for 60 seconds with a Peak Incidence Power of 50, Duty Factor of 10%, and 200 cycles per burst. Samples were run out on a gel to verify sonication success.

Library preparation followed the general protocol of Starrett et al. (2017) and the UCE website (ultraconserved.org), with some modifications. Briefly, libraries were prepared using the KAPA Hyper Prep Kit (Kapa Biosystems), using up to 250 ng DNA (i.e., half reaction of manufacturer’s protocol) as starting material. Ampure XP beads (Beckman Coulter) were used for all cleanup steps. For samples containing <250 ng total, all DNA was used in library preparation. After end-repair and A-tailing, 5 μM universal adapter stubs (University of Georgia, EHS DNA Lab) were ligated onto libraries. Libraries were then amplified in a 50 μl reaction, which consisted of 15 μl adapter-ligated DNA, 1X HiFi HotStart ReadyMix, and 0.5 μM of each Illumina TruSeq dual-indexed primer (i5 and i7) with modiﬁed 8-bp indexes (Glenn et al. 2016). Amplification conditions were 98 °C for 45 s, then 16 cycles of 98 °C for 15 s, 60 °C for 30 s, and 72 °C for 60 s, followed by a final extension of 72 °C for 60 s. Samples were quantified to ensure amplification success. Equimolar amounts of libraries were combined into 1000 ng pools consisting of eight samples each (125 ng per sample).

Target enrichment was performed on pooled libraries using the MYbaits Arachnida 1.1K version 1 kit (Arbor Biosciences) following the Target Enrichment of Illumina Libraries v. 1.5 protocol (http://ultraconserved.org/#protocols). Hybridization was conducted at 65 °C for 24 hours, then libraries were bound to streptavidin beads (Dynabeads MyOne C1, Invitrogen) and washed. Following hybridization, pools were amplified in a 50 μl reaction consisting of 15 μl of hybridized pools, 1X Kapa HiFi HotStart ReadyMix, 0.25 μM of each of TruSeq forward and reverse primers, and 5 μl dH20. Amplification conditions consisted of 98 °C for 45 s, then 16 cycles of 98 °C for 15 s, 60 °C for 30 s, and 72 °C for 60 s, followed by a final extension of 72 °C for 5 minutes. Following an additional cleanup, libraries were quantified using a Qubit fluorometer. Molarity was determined with an Agilent 2100 Bioanalyzer and equimolar mixes were prepared for sequencing on an Illumina NextSeq (University of California, Riverside Institute for Integrative Genome Biology) with 150 bp PE reads.

### Bioinformatic and phylogenomic analyses

Raw demultiplexed reads were processed entirely in the Phyluce pipeline (Faircloth 2015). Quality control and adapter removal were conducted with the Illumiprocessor wrapper (Faircloth 2013). Assemblies were created with Trinity r2013-02-25 (Grabherr et al. 2011) and Velvet 1.21 at default settings. For each sample, the fasta files from both assembly methods were combined into a single file. The combined assembly contigs were matched to probes using minimum coverage and minimum identity values of 65 with a modified version of the “phyluce_assembly_match_contigs_to_probes” script to allow multiple hits to a single probe. UCE loci were aligned with MAFFT (Katoh and Standley 2013) and trimmed with Gblocks (Castresana 2000, Talavera and Castresana 2007) with custom blocks settings (b1 = 0.5, b2 = 0.5, b3 = 6, b4 = 6) implemented in the Phyluce pipeline. Individual UCE alignments were imported into Geneious 11.0.4 (http://www.geneious.com, Kearse et al. 2012) and manually inspected for obvious alignment errors and to remove any potential non-homologous sequences. In this case, ingroup sequences more divergent than outgroup taxa based on pairwise genetic distance calculated in Geneious were flagged and removed as potential non-homologs. Two datasets were produced including loci at two different taxon coverage thresholds (50% and 70%). All bioinformatic analyses were performed on a late 2015 iMac, except for contig assembly, which was run on the University of California, Riverside Institute for Integrative Genome Biology Linux cluster.

Analyses of both 50% and 70% datasets included concatenated maximum likelihood, concatenated Bayesian, and coalescent-based analyses, while partitioned maximum likelihood analyses were run only on the 70% dataset with partitions and models determined by PartitionFinder v1.1.1 (Lanfear et al. 2012). Maximum likelihood trees were estimated with RAxML v8.2 (Stamatakis 2014) using the rapid bootstrap algorithm, 500 bootstrap replicates, and the GTRGAMMA model. Bayesian analyses were conducted using the BEAST v2.4 package (Bouckaert et al. 2014), run for 100 million generations, logging every 1000 generations, with 10% burnin. To assess convergence, Tracer (Rambaut et al. 2007) was used to check for ESS values >200 and examine stationarity of parameters. Two separate analyses were run to check for convergence between runs. All RAxML and BEAST analyses were run through the CIPRES PORTAL (Miller et al. 2010). To incorporate coalescent approaches, ASTRAL-II (Mirarab et al. 2014, Mirarab and Warnow 2015) was used with individual gene trees estimated in RAxML, and SVDQuartets (Chifman and Kubatko 2014, 2015) was run through PAUP* 4.0a159 (Swofford 2003), with 100 bootstrap replicates.

## Results

### Sequencing results

Sequencing results and data matrix statistics are presented in Suppl. material 1. Raw sequence reads are available in the NCBI Short Read Archive Accession no. SRP142540 (BioProject ID PRJNA451420). Untrimmed contigs, all trimmed individual locus alignments (pre- and post-manual editing), and trimmed concatenated matrices are available from the Dryad Digital Repository: http://dx.doi.org/10.5061/dryad.tj48997. Combining assemblies resulted in higher numbers of resulting UCE loci relative to using only a single assembly method, suggesting assembly-specific contigs as found in Hedin et al. (2018). The average number of UCE loci sequenced was 748 across all travunioid samples and 675 across all samples included in this study. The 70% taxon coverage matrix included 317 loci (272 average loci per sample, total length of 83,990 bp, 253.86 bp average locus length) and the 50% taxon coverage matrix included 677 loci (488 average loci per sample, total length of 165,096 bp, 264.95 average locus length).

### Phylogenomic analyses

**Figure 3.**
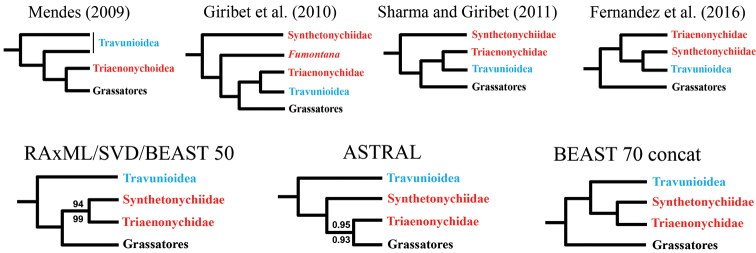
Phylogenetic relationships among major laniatorean lineages. Lower phylogenies correspond to results presented in this study. Nodes are fully supported (100% bootstrap or 1.0 posterior probability), unless indicated otherwise. Numbers in lower left phylogeny correspond to support values from 50% RAxML concatenated (top), and from 70% RAxML partitioned (bottom) analyses. Numbers in ASTRAL phylogeny based on 50% (top) and 70% (bottom) matrices.

All analyses, with the exception of the 70% concatenated BEAST analysis, recover Travunioidea as sister group to all other Laniatores lineages (Figure 3 and Suppl. material 2: Figure 1), a relationship not recovered in previous molecular phylogenetic studies. However, based on morphological data, this hypothesis has been put forth by Kury (2015) who created the name Tricospilata Kury, 2015 for Triaenonychoidea + Grassatores (the sister group to Travunioidea). The 70% concatenated BEAST analysis recovers Travunioidea + Triaenonychoidea Sørensen, 1886 (= Synthetonychiidae + Triaenonychidae), previously called Insidiatores Loman, 1900, sister group to the Grassatores. The only other phylogenetic analysis resulting in Insidiatores as sister group to Grassatores is the transcriptome-based study of Fernández et al. (2016). Synthetonychiidae is recovered as sister group to Triaenonychidae with full support in all RAxML and SVDQuartets analyses and the concatenated BEAST analysis, but is sister group to all non-travunioid Laniatores (Triaenonychidae + Grassatores) in the ASTRAL analyses. It is apparent that denser taxonomic sampling and further phylogenomic datasets will be required to resolve the base of Laniatores.

**Figure 4.**
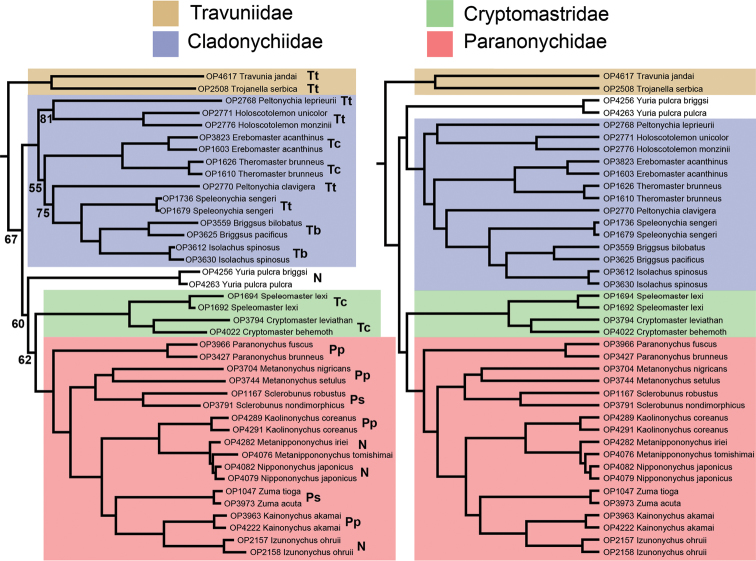
Phylogenomic relationships among travunioid genera. Left: RAxML and 50% BEAST concatenated topologies, with bootstrap support from the partitioned analysis. All nodes in the BEAST topology have posterior probability of 1.0. Abbreviations indicate placement in classification at the time of Kury et al. (2014): Tt = Travuniidae, Travuniinae; Tc = Travuniidae, Cladonychiinae; Tb = Travuniidae, Briggsinae; Pp = Paranonychidae, Paranonychinae; Ps = Paranonychidae, Sclerobuninae; N = Nippononychidae. Right: 70% BEAST concatenated. Nodes are fully supported (100% bootstrap or 1.0 posterior probability), unless indicated.

**Figure 5.**
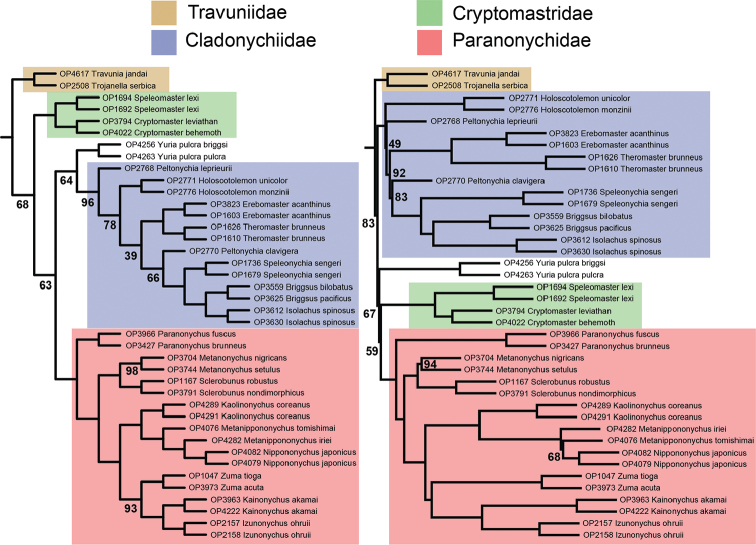
Phylogenomic relationships among travunioid genera. Left: 70% SVDQuartets. Right: 70% ASTRAL. Nodes are fully supported (100% bootstrap), unless indicated.

Travunioidea is monophyletic and fully supported across all analyses (Figure 3). Within Travunioidea, no families (and all but one subfamily) as currently defined in Kury et al. (2014) are monophyletic in any analyses (Figs 4, 5). A highly supported sister relationship between *Travunia* and *Trojanella* is recovered, and this group is sister to all remaining travunioids in all analyses. The western North American genera *Cryptomaster* Briggs, 1969 and *Speleomaster* Briggs, 1969 are recovered as sister taxa, and although the placement of *Cryptomaster* + *Speleomaster* is inconsistent across analyses, they are never sister group to or included within the Travuniidae or Cladonychiinae *sensu* Kury et al. (2014). In all analyses, the traditional Briggsinae (*Briggsus* Özdikmen & Demir, 2008 + *Isolachus* Briggs, 1971) are recovered within a largely travuniid clade, and always the sister group to *Speleonychia* Briggs, 1974. Eastern North American *Erebomaster* Briggs, 1969 + *Theromaster* Briggs, 1969 (traditional Cladonychiinae) is the sister group to the Briggsinae + *Peltonychia clavigera* (Simon, 1879) in all analyses. The two samples of the European genus *Peltonychia* Roewer, 1935 included in this study are never sister, *P. leprieurii* (Lucas, 1861) is found as the sister group to *Holoscotolemon* Roewer, 1915 while *P. clavigera* is the sister group to Briggsinae + *Speleonychia*. The relationships among these lineages are generally weakly supported at multiple nodes. All analyses recover a clade comprised of the former “northern triaenonychids”, currently in the families Paranonychidae and Nippononychidae, although neither family as currently defined is monophyletic. The Californian endemic genus *Zuma* Goodnight & Goodnight, 1942 is recovered within a clade comprised of Japanese taxa except *Yuria* Suzuki, 1964 and *Paranonychus fuscus* (Suzuki, 1976). The relationships within this lineage are all highly supported and identical across all analyses. The placement of the Japanese genus *Yuria* differs considerably across analyses and is recovered as the sister group to either the traditional travuniids or to a clade comprising *Cryptomaster* + *Speleomaster* and the Paranonychidae + Nippononychidae. Most importantly, *Yuria* is never recovered with the other Japanese nippononychids.

### Phylogenomic revision

**Figure 6.**
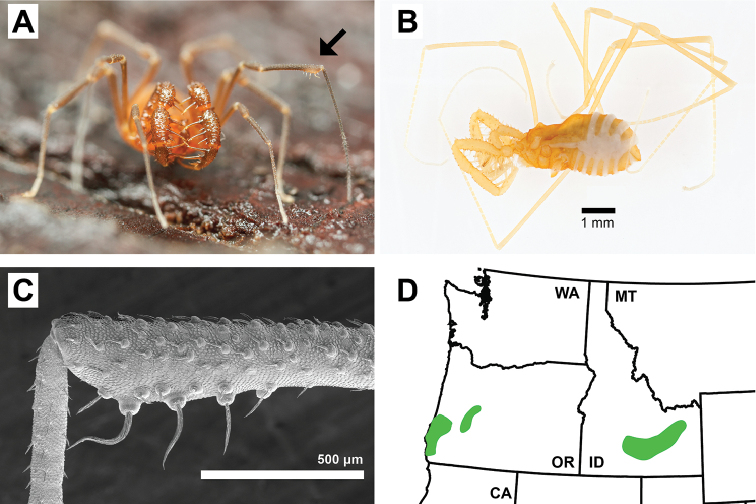
Morphology of Cryptomastridae. A) male *Cryptomaster*, arrow denotes sexually dimorphic swelling diagnostic of Cryptomastridae; B) *Speleomaster*, habitus; C) Scanning electron micrograph of tibial swelling, from Starrett et al. (2016); D) Cryptomastrid distribution.

Our approach to establish a stable classification involved identifying the largest group of terminal taxa that are always monophyletic and always highly supported across all analyses. We discovered four multi-genus clades consistent across all analyses (Figs 4, 5), treated here as families:

1) A clade containing *Travunia* + *Trojanella*. Because of the inclusion of *Travunia*, this clade retains the name Travuniidae.

2) A clade containing the majority of travuniid genera *sensu* Kury et al. (2014): *Peltonychia*, *Holoscotolemon*, *Erebomaster*, *Theromaster*, *Speleonychia*, *Briggsus*, and *Isolachus*. This clade will use the re-elevated and re-circumscribed familial name Cladonychiidae (see below).

3) A clade containing all genera currently included in the Paranonychidae and Nippononychidae of Kury et al. (2014). This clade retains the familial name Paranonychidae.

4) A clade consisting of the two former Cladonychiinae genera *Cryptomaster* and *Speleomaster*, endemic to the Pacific Northwest of North America, described below as the new family Cryptomastridae fam. n. (Figure 6).

The genus *Yuria* is considered *incertae sedis* given its uncertain phylogenetic placement (see Discussion). The new phylogenomics-based classification, used hereafter, is summarized in Table 2.

**Table 2.** Proposed revised classification. The number of described species/subspecies at each taxonomic level is in parentheses. Genera are grouped by phylogenetic affinity, not alphabetically.

Travunioidea (69/+11)	
**Travuniidae (6)**	**Cryptomastridae (4)**
*Dinaria* (1)	*Cryptomaster* (2)
*Travunia* (4)	*Speleomaster* (2)
*Trojanella* (1)	
**Cladonychiidae (30/+1)**	**Paranonychidae (28/+9)**
*Arbasus* (1)	*Paranonychus* (3)
*Buemarinoa* (1)	*Metanonychus* (3/+5)
*Peltonychia* (8)	*Sclerobunus* (12)
*Holoscotolemon* (8)	*Kaolinonychus* (1/+1)
*Erebomaster* (3/+1)	*Metanippononychus* (4/+2)
*Theromaster* (2)	*Nippononychus* (1)
*Briggsus* (5)	*Zuma* (2)
*Isolachus* (1)	*Kainonychus* (1/+1)
*Speleonychia* (1)	*Izunonychus* (1)
	**incertae sedis (1/+1)**
	*Yuria* (1/+1)

## Taxonomy

Below we redefine and diagnose all families of Travunioidea, including the newly described Cryptomastridae. The unsampled European genera are placed into two of these families based on previous morphological studies: *Dinaria* is placed in the Travuniidae with *Travunia* and *Trojanella*, while *Arbasus* and *Buemarinoa* are placed in the Cladonychiidae with *Peltonychia* and *Holoscotolemon*. It is premature to discuss definitive morphological synapomorphies for all travunioids, as all hypothesized members have never been surveyed for all relevant morphological characters. However, likely morphological synapomorphies include the presence of a four-lobed ovipositor and a bipartite intestinal *diverticulum tertium* (OD3 below; Dumitrescu 1975, 1976, reviewed in Giribet and Kury 2007).

### Abbreviations used for intestinal diverticula

**D1** diverticulum 1;

**OD2** opisthosomal diverticula 2;

**OD3** opisthosomal diverticula 3.

Terminology and homology for penis/glans structure follows Martens (1986).

### Suborder LANIATORES Thorell, 1876

#### Family CRYPTOMASTRIDAE

Taxon classificationAnimaliaOpilionesCryptomastridae

Derkarabetian & Hedin fam. n.

http://zoobank.org/A32A845F-36A7-426B-B3C1-F4E65085F356

##### Type genus.

*Cryptomaster* Briggs, 1969

##### Type species.

*Cryptomaster leviathan* Briggs, 1969

##### Diagnosis.

The Cryptomastridae can be diagnosed from all other travunioids by the presence of a distal swelling on tibia II that bears enlarged setae (Figure 6A, C), a sexually dimorphic structure found only in males. Both genera are fairly distinctive. *Cryptomaster* is easily identified as the largest (>2.5 mm body length) laniatorean in the Pacific Northwest of North America (Figure 6D) and largest member of Travunioidea, although two size forms exist (Starrett et al. 2016). *Speleomaster* species are restricted to lava tubes showing extreme levels of troglomorphy with complete absence of eyes, extremely reduced pigmentation, and leg elongation (Figure 6B). Although unrelated, *Speleomaster* and *Speleonychia* are both highly troglomorphic lava tube dwellers in the Pacific Northwest, found in Idaho and Washington, respectively. Aside from their disjunct geographic distribution, *Speleomaster* can be differentiated from *Speleonychia* by the absence of a free ninth tergite and lateral sclerites, and by the presence of bifurcating tarsal claws of the hind legs (*Speleonychia* with a peltonychium). The cryptomastrid genera can be distinguished from the eastern North American Cladonychiidae (*Erebomaster* + *Theromaster*) by the spination of the pedipalpal tarsus, previously noted by Briggs (1969, 1974). Cryptomastrids possess five prominent spines on the lateral margins of the pedipalpal tarsus, three on the prolateral margin and two on the retrolateral margin. *Erebomaster* and *Theromaster* possess three pairs of prominent lateral spines (in some *Theromaster*, the two apical retrolateral spines are fused at the base). The Cryptomastridae are unique in intestinal morphology, possessing a combination of an elongate and triangular DI (similar to *Briggsus* and *Isolachus*), and shorter OD2 and OD3 (similar to the Paranonychidae) (Suppl. material 2: Figure 2).

##### Included genera and species.

***Cryptomaster*.** Described by Briggs (1969) and originally included only *Cryptomaster leviathan* Briggs, 1969 from the Coastal Range of southwestern Oregon. A second species, *Cryptomaster behemoth* Starrett & Derkarabetian, 2016, was described from the west-central Cascade Range of Oregon (Starrett et al. 2016).

***Speleomaster*.** Briggs (1974) described the genus and both known species, *Speleomaster lexi* Briggs, 1974 and *Speleomaster pecki* Briggs, 1974, from lava tubes of the Snake River Plain in southern Idaho.

#### Family TRAVUNIIDAE

Taxon classificationAnimaliaORDOFAMILIA

Absolon & Kratochvíl, 1932

##### Type genus.

*Travunia* Absolon, 1920.

##### Type species.

*Travunia troglodytes* (Roewer, 1915).

##### Diagnosis.

It is difficult to diagnose the Travuniidae as all taxa have yet to be examined for all relevant characters. For all species in which male genitalia have been examined, the glans is widened and flattened with lateral extensions, tooth-like in *Trojanella* and wing-like in *Travunia* and *Dinaria*. The Travuniidae as defined here are restricted to the European Dinaric Karst and are highly troglomorphic, completely blind with a highly reduced ocularium (Figure 1F). The penis of *Travunia* and *Dinaria* is undifferentiated, while that of cladonychiids shows a clear division between glans and shaft. *Trojanella* shows some similarities in glans structure to *Holoscotolemon* and *Peltonychia* (e.g., divided glans and shaft), but the penis musculature is restricted to the apical portion of the shaft and glans in *Trojanella*, while the musculature of the European cladonychiids are restricted to the basal portion of the shaft.

##### Included genera and species.

***Travunia*.** The genus *Travunia* includes four described species that are all highly troglomorphic and restricted to caves in the southern Dinaric Karst region of Europe: *T. borisi* (Hadži, 1973) from Bosnia and Herzegovina, *T. hofferi* (Šilhavý, 1937) from Montenegro, *T. jandai* Kratochvíl, 1937 from Croatia, and *T. troglodytes* (Roewer, 1915) from Croatia and Bosnia and Herzegovina.

***Trojanella*** (Figure 1F). This monotypic genus is represented by *T. serbica* Karaman, 2005, a highly troglomorphic species restricted to a single cave on Stara Planina Mountain in Serbia.

***Dinaria*.** A monotypic genus represented by the highly troglomorphic species *D. vjetrenicae* (Hadži, 1932) known only from Vjetrenica Cave in southern Bosnia and Herzegovina.

##### Remarks.

It is not surprising that *Trojanella* is included in the most early-diverging travunioid lineage given Karaman’s (2005) statement that this species is a “unique and isolated phylogenetic line in the superfamily”. Karaman’s decision to leave *Trojanella* unplaced in Travunioidea was made to highlight, and is a consequence of, the commonly used morphological characters that have hindered a reliable taxonomy within this group. It is unclear how many species of *Travunia* actually exist. Novak (2004) questioned the validity of *Travunia*, *Dinaria*, and *Abasola* at the generic level, and *Abasola* was later synonymized with *Travunia* (Kury and Mendes 2007). Novak (2004, 2005) argues that *Travunia* may be oversplit and includes only 2–3 species of questionable status. The status of *Travunia* and *Dinaria* as distinct genera has been questioned based on similarity in male genitalic morphology (Novak 2004); together these might represent a single lineage.

#### Family CLADONYCHIIDAE

Taxon classificationAnimaliaORDOFAMILIA

Hadži, 1935

##### Type genus.

*Erebomaster* Briggs, 1969.

##### Type species.

*Erebomaster flavescens* Cope, 1872.

##### Diagnosis.

**Figure 7.**
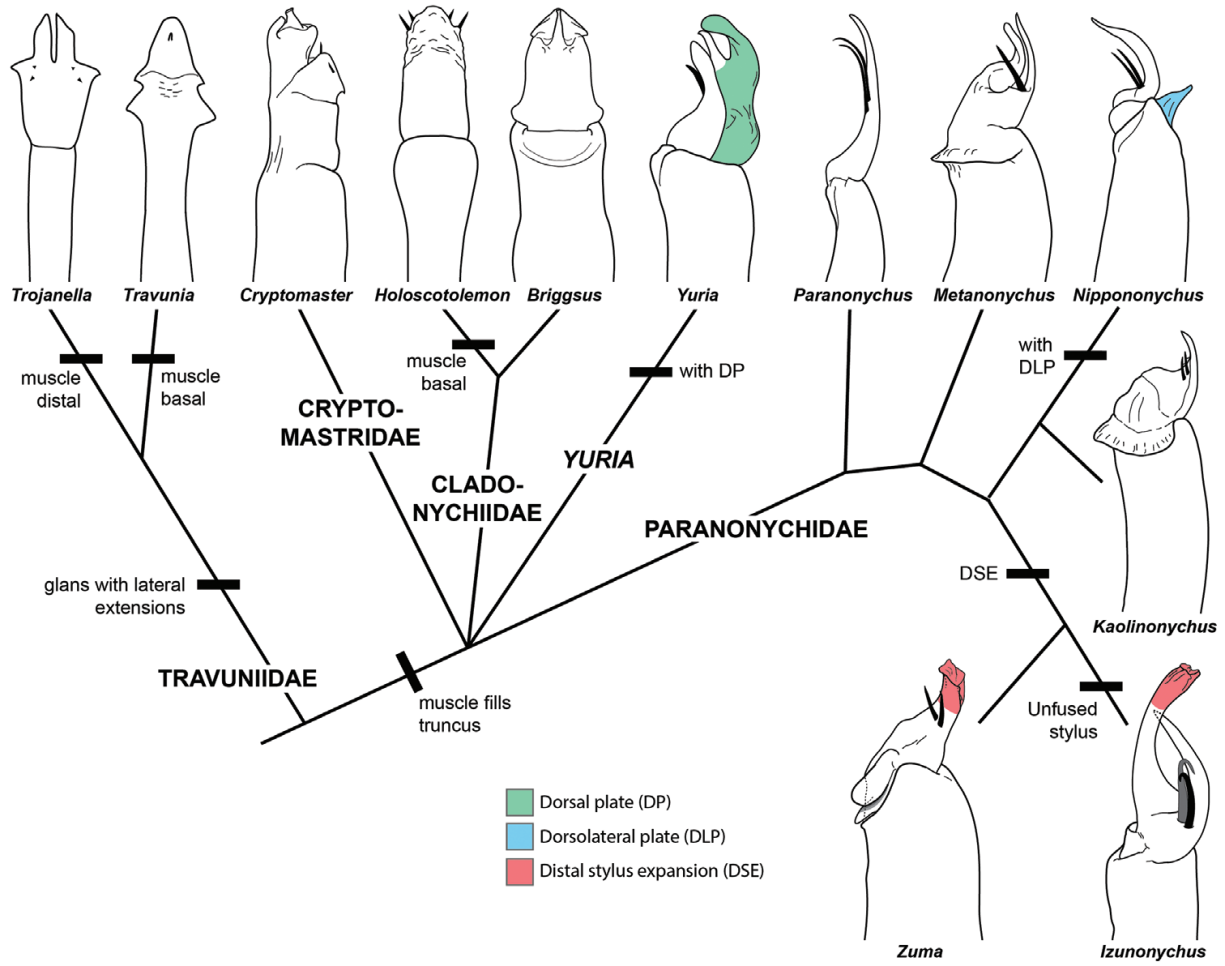
Representative penis morphology of Travunioidea. Clockwise from left: *Trojanella serbica* redrawn from Karaman (2005), *Travunia hofferi* redrawn from Karaman (2005), *Cryptomaster behemoth* adapted from Starrett et al. (2016), *Holoscotolemon jaqueti* redrawn from Martens (1978), *Briggsus hamatus*, *Yuria pulcra*, *Paranonychus brunneus*, *Metanonychus setulus navarrus*, *Nippononychus japonicus* redrawn from Suzuki (1975), *Kaolinonychus coreanus coreanus* redrawn from Suzuki (1975), *Izunonychus ohruii*, *Zuma acuta*. All Travuniidae and Cladonychiidae are drawn in ventral view; Cryptomastridae, *Yuria*, and Paranonychidae drawn in lateral. For simplicity, not all travunioid genera are included.

Some taxa have not been examined for the relevant characters, but tentative diagnostic characters may be found in the intestinal complex (Suppl. material 2: Figure 2). All Cladonychiidae that have been examined show a 2–3 branched, elongate, and triangular D1, and elongate OD3. In the Pacific Northwest of North America, cladonychiids are broadly sympatric with the Cryptomastridae and Paranonychidae. The above intestinal characteristics differentiate them from Cryptomastridae, which possess a relatively short and stout OD3, and the Paranonychidae, which possess a simple unbranched D1 (Suppl. material 2: Figure 2). The European taxa can be diagnosed from Travuniidae based on male genital morphology (Figure 7): travuniids have a widened and flattened glans with lateral wing-like extensions; the glans and shaft are undivided in *Travunia* and *Dinaria*. The penis musculature is restricted to the base in *Holoscotolemon* and *Peltonychia*, while the musculature of *Trojanella* is restricted to the apical portion of the shaft and glans.

##### Included genera and species.

***Erebomaster*** (Figure 1B). *Erebomaster* is found in the eastern United States, and currently includes three species: *E. flavescens* Cope, 1872 with two subspecies *E. f. flavescens* from Wyandotte Cave in Indiana and *E. f. coecus* (Packard, 1888) from Carter Cave in Kentucky; *E. weyerensis* (Packard, 1888) from caves in West Virginia; and the relatively widespread *E. acanthinus* (Crosby & Bishop, 1924) with a distribution along and west of the Appalachian Mountains. A revision of *Erebomaster* taxa is needed.

***Theromaster*** (Figure 1A). Consisting of two described species found in the eastern United States: *T. brunneus* (Banks, 1902) is relatively widespread in the southern Appalachian Mountains; *T. archeri* (Goodnight & Goodnight, 1942) from caves in Alabama.

***Speleonychia*** (Figure 1I). A monotypic genus, *Speleonychia sengeri* Briggs, 1974 is a highly troglomorphic species restricted to lava tubes near Mt. Adams, in south-central Washington.

***Briggsus*** (Figure 1G). The genus and all species were originally described by Briggs (1971b) as *Pentanychus*; Özdikmen and Demir (2008) provided the replacement name. This genus consists of five described species all restricted to the moist coastal forests (>50 inches yearly rainfall) of Oregon and Washington in the Pacific Northwest: *B. bilobatus* (Briggs, 1971), *B. clavatus* (Briggs, 1971), *B. flavescens* (Briggs, 1971), *B. hamatus* (Briggs, 1971), and *B. pacificus* (Briggs, 1971).

***Isolachus*** (Figure 1H). A monotypic genus, *Isolachus spinosus* Briggs, 1971 is restricted to northwest Oregon and southwest Washington.

***Holoscotolemon*** (Figure 1D). A European genus with eight species. Six species are restricted to the Alps, primarily from Italy and Austria: *H. unicolor* Roewer, 1915; *H. lessiniensis* Martens, 1978; *H. oreophilus* Martens, 1978; *H. franzinii* Tedeschi & Sciaky, 1994; *H. monzinii* Tedeschi & Sciaky, 1994; and *H. naturae* Tedeschi & Sciaky, 1994. *H. querilhaci* (Lucas, 1864) is found in the Pyrenees of southern France and *H. jaqueti* (Corti, 1905) is recorded from eastern Europe in Romania, Ukraine, and former Yugoslavia.

***Peltonychia*** (Figure 1E). A genus with a long history, *Peltonychia* includes the first described travunioid species, *P. leprieurii*. This genus of eight species is almost entirely known only from caves in central Europe throughout the Pyrenees and Alps (Suppl. material 2: Figure 3). *Peltonychia leprieurii* is found in the Alps of northern Italy. *Peltonychia clavigera*, *P. navarica* (Simon, 1879), *P. piochardi* (Simon, 1872), and *P. sarea* (Roewer, 1935) are all found in the Pyrenees of northern Spain and southern France. *P. gabria* Roewer, 1935 is recorded from Trieste, Italy; *P. postumicola* (Roewer, 1935) is recorded from eastern Italy and western Slovenia; and *P. tenuis* Roewer, 1935 is recorded from northern Slovenia. Records from Trieste, Italy and Slovenia are conclusively shown to be in error, and *P. postumicola* and *P. tenuis* are morphologically similar to *P. clavigera* (Novak and Gruber 2000). As such, it is unclear how many actual species are included.

***Arbasus*.** A monotypic genus, the highly troglomorphic *Arbasus caecus* (Simon, 1911) is only known from Grotte de Pène Blanque in the Pyrenees of southern France.

***Buemarinoa*.** A monotypic genus, the highly troglomorphic *Buemarinoa patrizii* Roewer, 1956 is only known from the Grotte del Bue Marino in Sardinia, Italy.

***Proholoscotolemon*** Ubick & Dunlop, 2005. A monotypic genus, *P. nemastomoides* (Koch & Berendt, 1854) is known from specimens preserved in Baltic amber. The specimens were redescribed by Ubick and Dunlop (2005) and based on morphological similarity and geography it is interpreted as the ancestor of, or sister group to, *Holoscotolemon*.

##### Remarks.

*Peltonychia* is polyphyletic, in some cases with strong support (Figure 5). The sampled species are from two separate geographic regions: *P. clavigera* from the Pyrenees of northern Spain and southern France, and *P. leprieurii* from the Alps of northern Italy. Accounting for the locality errors in Italy and Slovenia mentioned above, *Peltonychia* is geographically split into two regions: *P. leprieurii* in northern Italy, and the remaining species in the Pyrenees. The male genitalia of four species of *Peltonychia* have been examined: *P. leprieurii*, *P. clavigera*, *P. gabria*, and *P. postumicola*. Based on these genitalic drawings (Chemini 1985, Martens 1978, Thaler 1996), it is obvious that *P. leprieurii* is very divergent from the other three *Peltonychia*, which are very similar (Suppl. material 2: Figure 3). This concordance between geography, genital morphology, and our phylogenomic analyses support the separation of *Peltonychia* into two genera. However, we refrain from formally making this taxonomic change until all relevant species can be studied.

The sister relationship of *Speleonychia* to the traditional Briggsinae (*Briggsus* + *Isolachus*) is not surprising given the close geographic proximity of these genera and shared presence of a free ninth tergite and lateral sclerites. The distinct generic status of *Arbasus* and *Buemarinoa* has been doubted (Kury and Mendes 2007). The morphological distinction between *Arbasus* and *Buemarinoa* is minimal and entirely based on tarsal segmentation (Kury and Mendes 2007), which is typical of the “Roewerian classification” system that resulted in taxa being over split based on irrelevant characters (e.g., Kury et al. 2014, Kury and Pérez-González 2015). Aside from the original descriptions with basic drawings (Roewer 1935, 1956), virtually no taxonomic work has been conducted on *Arbasus* and *Buemarinoa*. However, Kury and Mendes (2007) note that they “both look superficially like *Hadziani* [=*Peltonychia*], but with clear troglomorphic traits…”, and their inclusion in Cladonychiidae here seems justified.

#### Family PARANONYCHIDAE

Taxon classificationAnimaliaORDOFAMILIA

Briggs, 1971

##### Type genus.

*Paranonychus* Briggs, 1971

##### Type species.

*Paranonychus brunneus* (Banks, 1893).

##### Diagnosis.

The Paranonychidae can be diagnosed by their relatively complex glans (except *Paranonychus*) (Figure 7 and Suppl. material 2: Figure 4), and by their intestinal complex (Suppl. material 2: Figure 2). For all taxa that have been examined, the paranonychids possess a small D1 that is circular to subtriangular, and a simple and shorter OD3. The paranonychids are restricted to western North America and East Asia. In southern Japan the paranonychids are sympatric with *Yuria* and can be diagnosed by several characteristics: *Yuria* possesses a free ninth tergite, and the penis has a dorsal plate with fused stylus; the paranonychids do not have a free ninth tergite and the penis glans lacks a dorsal plate. In western North America, the paranonychids are sympatric and syntopic in surface habitats with the Cladonychiidae (*Briggsus*, *Isolachus*) and Cryptomastridae (*Cryptomaster*). The paranonychids can be differentiated from these families by the structure of D1: paranonychids possess a small circular to subtriangular unbranched D1, while Cladonychiidae and Cryptomastridae possess an elongate, triangular, and branched D1.

##### Included genera and species.

***Paranonychus*** (Figure 1K). This trans-Beringian genus includes three known species: *P. brunneus* (Banks, 1893) distributed in the Coast and Cascade Ranges of Oregon and Washington with records extending north to Alaska; *P. concolor* Briggs, 1971, recorded from a single location in the southern Cascade Range of Oregon; and *P. fuscus* found throughout northern Honshu in Japan.

***Metanonychus*** Briggs, 1971. This genus and all species were described by Briggs (1971) and are restricted to the moist forests of the Pacific Northwest of North America. *Metanonychus* includes three species: *M. nigricans* Briggs, 1971 with two subspecies, *M. n. nigricans* and *M. n. oregonus*, found in Oregon; *M. setulus* Briggs, 1971 with five subspecies, *M. s. setulus*, *M. s. cascadus*, *M. s. mazamus*, *M. s. navarrus*, and *M. s. obrieni*, found in Oregon, Washington, and northern California; and *M. idahoensis* Briggs, 1971 found in northern Idaho.

***Sclerobunus*** Banks, 1893 (Figure 1L). Recently revised by Derkarabetian and Hedin (2014), *Sclerobunus* is distributed throughout western North America and currently includes 12 species divided into three species groups. The *nondimorphicus* group includes *S. nondimorphicus* Briggs, 1971 from Oregon, Washington, and British Columbia, and *S. idahoensis* Briggs, 1971 from northern Idaho. The cave-obligate *cavicolens* group includes: *Sclerobunus cavicolens* (Banks, 1905) restricted to Lewis and Clark Caverns, Montana; *Sclerobunus ungulatus* (Briggs, 1971) from caves in Great Basin National Park, Nevada; *Sclerobunus madhousensis* (Briggs, 1971) from caves near Provo, Utah. The *robustus* group includes the widespread *S. robustus* (Packard, 1877), *S. glorietus* Briggs, 1971, and *S. skywalkeri* Derkarabetian & Hedin, 2014, all distributed throughout the high elevation forests of the southwestern United States, and *S. jemez* Derkarabetian & Hedin, 2014, *S. klomax* Derkarabetian & Hedin, 2014, *S. speoventus* Derkarabetian & Hedin, 2014, and *S. steinmanni* Derkarabetian & Hedin, 2014, which are all troglomorphic species restricted to cave and talus habitats along the eastern edge of the southern Rocky Mountains in New Mexico and Colorado.

***Kaolinonychus*** Suzuki, 1975. This monotypic genus endemic to South Korea is recorded mostly from caves. *Kaolinonychus coreanus* (Suzuki, 1966) includes two subspecies *K. c. coreanus* and *K. c. longipes*.

***Metanippononychus*** Suzuki, 1975. (Figure 1M). Endemic to Japan, *Metanippononychus* is restricted to southern Honshu, Shikoku, and Kyushu and includes four species: *M. daisenensis* Suzuki, 1975; *M. iriei* Suzuki, 1975, with two subspecies *M. i. iriei* and *M. i. yakuensis*; *M. iyanus* Suzuki, 1975; *M. tomishimai* Suzuki, 1975, with two subspecies *M. t. tomishimai* and *M. t. awanus*.

***Nippononychus*** Suzuki, 1975. A monotypic genus endemic to Japan, *Nippononychus japonicus* (Miyosi, 1957) is restricted to southern Honshu and Shikoku.

***Zuma*** (Figure 1N). *Zuma* includes two species restricted to forests of central and northern California: *Zuma acuta* Goodnight & Goodnight, 1942 restricted to the coastal forests south of San Francisco; *Zuma tioga* Briggs, 1971 found in the central and northern Sierra Nevada range.

***Izunonychus*** Suzuki, 1975. A monotypic genus endemic to Japan, *Izunonychus ohruii* Suzuki, 1975 is restricted to the Izu peninsula and Hakone area in central Honshu.

***Kainonychus*** Suzuki, 1975 (Figure 1O). A monotypic genus endemic to Japan, *Kainonychus akamai* (Suzuki, 1972) includes two subspecies, *K. a. akamai* distributed throughout northern Honshu and *K. a. esoensis* restricted to Hokkaido.

##### Remarks.

In this study all genera in the Paranonychidae have been sampled and the generic relationships are consistent and highly supported across all analyses (Figs 4, 5). Although the study of Derkarabetian et al. (2010) only included North American taxa, the relationships of paranonychids recovered here are the same, notably *Paranonychus* as the earliest diverging genus, and a sister relationship between *Sclerobunus* and *Metanonychus*. The familial name Sclerobunidae has been used previously (Giribet et al. 2010) for the “northern triaenonychids”. However, Paranonychidae and the subfamily Paranonychinae Briggs, 1971 have priority over the names Sclerobunidae and Sclerobuninae Dumitrescu 1976.

The Japanese genera *Metanippononychus* and *Nippononychus* show levels of UCE divergence consistent with congeners (Figs 4, 5). Intermediate morphological forms between *Nippononychus japonicus* and *Metanippononychus daisenensis* can be found where the two species come into contact (Tsurusaki pers. obs.). These genera are differentiated only by tarsal claw structure: *Metanippononychus* possessing a ventral tooth on the median prong of the hind claws. The original drawings of male genitalia show that *M. daisenensis* and *N. japonicus* differ in the width of the stylus (Suzuki 1975b). However, the penis of *N. japonicus* is highly similar to that of the geographically proximate *M. tomishimai tomishimai*.

Kury et al. (2014) includes *Paranonychus fuscus* (formerly *Mutsunonychus fuscus*) as a synonym of *Paranonychus brunneus* (Banks, 1893) based on Shear’s (1986) statement “*Paranonychus brunneus* (=*Mutsunonychus fuscus* Suzuki; Paranonychidae)”. Later in Shear and Derkarabetian (2008), the genus *Mutsunonychus* was formally synonymized under *Paranonychus*, and although a potential species level synonymy was noted, it was not formally established. The levels of UCE divergence between *P. brunneus* and *P. fuscus* are consistent with species level divergences compared to other pairs of congeneric taxa included (Figs 4 and 5), and as such, *P. fuscus* is again treated as a distinct species here.

### Incertae sedis

**Included genera and species. *Yuria*** (Figure 1J). A monotypic genus endemic to Japan, *Yuria pulcra* Suzuki, 1964 includes two subspecies distributed throughout southern Honshu, Shikoku, and Kyushu: *Y. p. pulcra* and *Y. p. briggsi* Suzuki, 1975.

**Remarks.** When *Yuria pulcra* was first described it was placed in Travuniidae because the tarsal claw is a peltonychium (Suzuki 1964, 1975a). Phylogenetic analysis of morphological data placed *Yuria* as the sister group to *Nippononychus* (Paranonychidae) with two synapomorphies (Mendes 2009). Kury et al. (2014) later transferred this genus to the Nippononychidae, which contained most of the other Japanese travunioids. Placement and support for *Yuria* varies depending on analysis (Figs 4, 5). Morphology complicates matters further, as *Yuria* possesses a free ninth tergite and lateral sclerites, plesiomorphic characters that are potentially neotenic and shared with the Briggsinae. The penis morphology of *Yuria* is also relatively unique within Travunioidea, possessing a dorsal plate with fused stylus (Figure 7 and Suppl. material 2: Figure 4).

## Discussion

### Travunioidea classification and the trouble with travuniids

Travunioidea includes 80 nominal taxa (species/subspecies), four families, and one unplaced genus. Traditionally within Travunioidea the subfamilial rank has been used to further subdivide taxa, and the composition of subfamilies has changed across classification schemes (Table 1). Here, we refrain from using the subfamilial rank for three reasons: 1) the confusing taxonomic history of these lineages, specifically with regards to relative rank and composition; 2) the poorly supported nodes in Cladonychiidae and non-monophyly of *Peltonychia*; 3) and the relatively sparse composition each subfamily would have (i.e., 4/6 subfamilies would contain only 1–2 genera). Based on phylogenomic analyses, the composition of all subfamilies would have changed again (Table 2). Relationships among several traditional Travuniidae genera are still uncertain, and the absolute stability of the familial rank will be dependent upon future incorporation of the unsampled European genera *Arbasus* and *Buemarinoa*.

The traditional Travuniidae have had an incredibly long and complex taxonomic history beginning with the description of the first travunioid in 1861. Kury and Mendes (2007) focus entirely on nomenclatural issues, resolving them at the familial and generic level, and in doing so note that “the [traditional] family Travuniidae constitutes one of the worst problems of the laniatorid taxonomy of the 20^th^ century”. Several others have discussed the diverse array of problems plaguing travunioid taxonomy (Novak 2005, Novak and Gruber 2000, Karaman 2005). These issues include, but are not limited to, description of two species in two different genera based on the same material, autosynonymy of a genus name, proposal of unavailable family and genus names, genus description without designation of type species after 1930, species descriptions based on juveniles, mistranslation of foreign languages, disregard for correct taxonomic changes, mismatched type localities, type localities accidentally and intentionally incorrectly named, inability to find further specimens from type localities, and destruction of type localities. Even though the European taxa have received significant attention from a taxonomic standpoint, a great deal of focused and devoted research including fieldwork, and modern morphological and phylogenetic analyses will be needed to fully resolve the taxonomic issues of this notoriously difficult clade.

The goal of this research was to provide a stable classification of Travunioidea at the familial level. This stability relies on incorporating potential future changes if unsampled taxa are included. We believe our familial level classification, disuse of subfamily rank, and leaving *Yuria* unplaced, minimizes future taxonomic changes. All familial name-holding genera are included. Only *Arbasus* and *Buemarinoa* are missing but given morphological similarities and the geographic distribution of these taxa, their inclusion in Cladonychiidae given future sampling seems likely. The stability of the familial level provided by this phylogenomics-based reclassification and the recovered distinction between Travuniidae and European Cladonychiidae can guide future efforts. The morphologically enigmatic *Yuria* remains phylogenetically elusive. A potential solution to the unreliable placement of *Yuria* is to create a monotypic family. However, we refrain from this until all genera can be included in phylogenomic analyses.

### Morphological reevaluation

***The tarsal claw*** – A type of modified tarsal claw termed a peltonychium united the “traditional Travuniidae”, a structure now known to be convergent in several unrelated cave-dwelling taxa (e.g., *Peltonychia*, *Speleonychia*, *Trojanella*). The morphological distinction between the typical trident-shaped tarsal claw (with variable number of side-branches) and a peltonychium is not entirely clear in some travunioids (e.g., *Izunonychus*, *Metanippononychus*), and the transition between forms is best documented in the triaenonychid genus *Lomanella* Pocock, 1902 (Hunt and Hickman 1993). However, *all* of the 18 travunioid species (not including subspecies) with a clear peltonychium are found in caves, 10 of which are either described to be troglobitic (cave-obligate) and/or show high levels of troglomorphy. An additional six species are described to have reduced pigmentation (relative to surface-only species), five of which are only reported from caves. Two remaining species are inconsistent with this pattern. First, *Peltonychia leprieurii*, from the Alps of northern Italy and Switzerland is recorded from both cave and surface habitats (considered a troglophile) and retains black pigment. Second, *Y. pulcra pulcra* and *Y. p. briggsi* from southern Japan show some reduced pigmentation, though they differ in their habitat: *Y. p. pulcra* is recorded from both cave and surface habitats, while *Y. p. briggsi* is known only from surface habitats under woody debris. It has been suggested that the peltonychium, and other plesiomorphic characters, are convergent through neoteny (Rambla 1980, Hunt and Hickman 1993, Karaman 2005). The hind tarsal claws of juvenile triaenonychids and travunioids have more side branches than adults (e.g., Hunt and Hickman 1993, Suzuki 1975b), and some juveniles additionally possess a pseudonychium (median tarsal claw) (Shultz and Pinto-da-Rocha 2007, Gnaspini 2007). As such, the peltonychium may be a retained adult form of this juvenile structure (Hunt and Hickman 1993).

***The ninth tergite and lateral sclerites*** – The traditional Briggsinae (*Briggsus* + *Isolachus*) were hypothesized to be a relatively early diverging lineage within Travunioidea (Briggs 1971, Giribet and Kury 2007). This was due to the presence of a free ninth tergite, and lateral sclerites, a plesiomorphic condition found in the other suborders of harvestmen. Conversely, penis morphology suggested that this group is derived given the relatively simple penis structure (Martens 1986). *Speleonychia* also possess a distinct ninth tergite and lateral sclerites, now shown to be a shared condition with *Briggsus* + *Isolachus*. Rambla (1980) argued that these characters are neotenic, retained in adults from nymphal stages, and as such are derived. All phylogenomic analyses here support the derived nature of these characters, as they are recovered well within the Cladonychiidae in all analyses. However, not all travunioid taxa with a free ninth tergite and free lateral sclerites are restricted to this clade, as *Yuria* also possess both characters (Suzuki 1964, 1975a). While the placement of *Yuria* is uncertain, it is never recovered with or as the sister group to the traditional Briggsinae + *Speleonychia*. Outside of Travunioidea, the only other laniatorean taxa known to possess free lateral sclerites are in the genus *Hickmanoxyomma* Hunt, 1990, a largely cave-dwelling triaenonychid genus endemic to Tasmania (Hunt 1990). In this genus the presence of five pairs of free lateral sclerites (2–3 in travunioids) are a diagnostic character for the *H. cavaticum* species group containing four species, all recorded only from caves and some showing troglomorphy. Hunt (1990) suggested it might be correlated with the overall reduced sclerotization associated with troglomorphy, but also reiterated Rambla’s (1980) view of neoteny. No clear phylogenetic, evolutionary, or ecological pattern exists for these characters and their presence in multiple unrelated lineages suggests their plesiomorphic nature.

***The midgut*** – Studies focusing on the digestive tract began in the 1920s, but the work of Dumitrescu (e.g., 1974, 1975, 1976) contributed most significantly to the phylogenetic utility of midgut morphology in Opiliones. Through examination of intestinal morphology Dumitrescu (1975, 1976) noticed the “northern triaenonychids” are more similar to the Travuniidae as they share a bipartate OD3, instead of the southern hemisphere triaenonychids (3-branched). As such, he placed the “northern triaenonychid” taxa into the family Paranonychidae Briggs, 1971. The name Paranonychidae was used by a few subsequent authors (e.g., Shear 1982, 1986, Ubick and Dunlop 2005), but from a classification standpoint, was not incorporated into the taxonomy as the taxa were left in the Triaenonychidae (e.g., Kury 2003a, Pinto-da-Rocha and Giribet 2007; Kury 2007b). Later, molecular phylogenetic studies also showed the “northern triaenonychids” being grouped with the travunioid families (Giribet et al. 2010, Hedin and Thomas 2010, Derkarabetian et al. 2010, Sharma and Giribet 2011), and the familial name Paranonychidae was finally included in Opiliones taxonomy by Kury (2013).

Our phylogenomic analyses allow for a reexamination of the intestinal morphology research of Dumitrescu (1975, 1976) (Suppl. material 2: Figure 2). First, the recovery of Travunioidea as the earliest diverging Laniatores lineage is reflected in the branching pattern of OD3. All Travunioidea possess an OD3 with two branches, a characteristic shared with the other harvestmen suborders, while the Synthetonychiidae, Triaenonychidae, and Grassatores possess three branches. This phylogenetic utility of OD3 was also noted in the morphological analyses of Mendes (2009), who recovered Travunioidea as the earliest diverging lineage of Laniatores. The intestinal complex also shows some phylogenetic value within Travunioidea; three of four families can be differentiated entirely based on intestinal morphology. Of the taxa examined by Dumitrescu (1975, 1976), the paranonychids possess a simple and circular D1 with no branches, while all cladonychiid genera possess a relatively complex D1 with 2–3 branches or is distinctly triangular in shape. Karaman (2005) also reports a D1 with two branches for *Trojanella*. Cryptomastridae possess a triangular D1, but the shorter OD2 and OD3 are more similar to those of the paranonychids. Samples of *Briggsus* and *Speleonychia* were included and look very similar to each other, particularly the D1, which is recorded to be 3-branched in *Briggsus* and triangular in *Speleonychia* (Dumitrescu 1976). Dumitrescu did not note the similarity, instead stating *Speleonychia* was most similar to *Nippononychus japonicus* (then *Peltonychia japonica*, placed in Travuniidae), perhaps subjectively limited by the classification system of the time. It is clear that there is phylogenetic utility in midgut morphology at higher taxonomic levels, and further descriptions are needed to confirm the consistency of diagnostic characteristics noted here.

***The penis*** – Based on descriptions and drawings, the musculature and glans complexity can be used to diagnose and differentiate travunioid lineages recovered here (Figure 7) (Briggs 1971b, Suzuki 1975a, 1975b, Martens 1986, Karaman 2005, Novak 2005, Pinto-da-Rocha and Giribet 2007, Derkarabetian and Hedin 2014, Starrett et al. 2016). In Travunioidea, the glans is relatively simple and plate-like without dorsal, dorsolateral, or ventral plates in *Trojanella*, *Travunia*, Cryptomastridae, and Cladonychiidae, while in *Yuria* and Paranonychidae (except *Paranonychus*) the glans is relatively complex, and some possess a dorsolateral plate. The dorsal plate is absent from all travunioids, except *Yuria*, which possesses a dorsal plate that is fused to the stylus.

Given the consistent and highly supported relationships within Paranonychidae, diagnostic differences of paranonychid lineages can be seen in penis morphology (Figure 7; Suppl. material 2: Figure 4). Within Paranonychidae, the earliest-diverging genus *Paranonychus* possesses the simplest glans. Dorsolateral plates are present but reduced in two pairs of sister genera *Sclerobunus* + *Metanonychus* and *Metanippononychus* + *Nippononychus* (Briggs 1971b, Suzuki 1975b, Derkarabetian and Hedin 2014). Additionally, *Sclerobunus* + *Metanonychus* show a distinct setae-bearing process similar to a ventral plate (“sensillenträger” of Martens) that is fused to the base of the stylus in the *Zuma* + Japanese taxa. The *Zuma* + *Izunonychus* + *Kainonychus* clade possesses a modified stylus that is expanded distally (Briggs 1971b, Suzuki 1975b). As opposed to other Japanese paranonychids which have a stylus that is fused to the ventral plate, the stylus of both *Izunonychus* + *Kainonychus* is separated from the ventral plate (Suzuki 1975b).

The overall trend across Opiliones suborders is one of apparently increasing genitalic complexity. The earliest-diverging suborder Cyphophthalmi has a spermatopositor, the Dyspnoi and Eupnoi have a simple penis with relatively little modifications, and the derived Laniatores possess the most complex penes (Macías-Ordóñez et al. 2010). Within Laniatores the trend of increasing complexity is maintained, as Travunioidea with relatively simple glans morphology is possibly the most early-diverging Laniatores. Travunioidea and Triaenonychoidea use muscles for glans expansion (Shultz and Pinto-da-Rocha 2007, Pérez-González and Werneck 2018), a condition shared with Eupnoi and Dyspnoi. Relative to travunioids, the synthetonychiids and triaenonychids have slightly more complex glans structures with dorsolateral plates. Finally, in the most diverse laniatorean lineage Grassatores, muscles are absent, and the glans requires hydraulic pressure to expand (Shultz and Pinto-da-Rocha 2007). In Travunioidea, Martens (1986) noted a tendency towards simplification of the glans, and Karaman (2005) additionally noted a correlation where simplification of the glans structure is associated with reduction of penis musculature to basal portion of the truncus. Both Martens and Karaman argued that the simple glans of travunioids (i.e., traditional travuniids) is a derived condition, as all Triaenonychoidea have relatively complex glans with dorsolateral plates and a distinct process bearing setae, two characters lost in many travunioids. Most phylogenomic analyses conducted here recover Travunioidea as the most early-diverging Laniatores lineage suggesting a trend of simple to complex penis structure. It is interesting to note that the simplest glans and some taxa with relatively reduced musculature are found in cave-inhabiting taxa (e.g., *Trojanella*, *Dinaria*, *Travunia*), which may be a potential confounding factor in establishing a clear evolutionary trend. More detailed morphological examinations of the male and female genitalia using modern approaches (e.g., Pérez-González and Werneck 2018) will help elucidate evolutionary trends and provide more diagnostic characters for the families established here.

### Context for future research

This phylogenomic study provides a more stable taxonomy for Travunioidea, which serves as a starting point for species-level phylogenomics and provides the phylogenetic context to explore evolutionary questions relating to character evolution, alpha taxonomy, and biogeography.

***Morphological and chemical evolution*** – Many Travunioidea are cave-obligate taxa with species from 14 genera showing some degree of troglomorphy, possessing homoplastic morphological features that evolve as a response to cave life. Travunioidea can be an excellent system to study the repeated evolution of troglomorphy, as it has evolved at multiple taxonomic levels (e.g., within families, genera, species) with multiple independently derived taxa showing varying degrees of troglomorphy. For example, within the genus *Sclerobunus*, troglomorphy has evolved at least five times independently across multiple species and within single species and is time-correlated (Derkarabetian et al. 2010, Derkarabetian and Hedin 2016). A species-level phylogeny for all travunioids would allow for an accurate estimate of the number of independent evolutions of troglomorphy and allow for in-depth morphological analyses exploring the rate and timing of this potentially adaptive morphology, as well as providing the phylogenetic framework for comparative studies (e.g., gene expression).

Similarly, chemical evolution can be explored in this phylogenomic context. Harvestmen possess repugnatorial glands, which are used to store chemical cocktails that are secreted in defensive behavior. Chemical composition across lineages has been shown to have some phylogenetic value, particularly in Laniatores (Raspotnig 2012, Raspotnig et al. 2015). Similarly, a study focusing on Travunioidea and Triaenonychoidea has shown high levels of divergence in chemical composition between taxa formerly united under the traditional taxonomy (i.e., Cladonychiidae) (Shear et al. 2014). Given the phylogenetic context, detailed chemical analyses can be used to discover novel chemicals, compare pathways of identical chemicals found in independent lineages, and explore the evolution of biochemical pathways across taxa (e.g., Rodriguez et al. 2018).

***Biogeography and alpha taxonomy*** – Cryophilic harvestmen are useful in biogeographic analyses because of restricted ecological constraints and extremely low vagility. Previous molecular phylogenetic studies on harvestmen with these biological characteristics have shown compelling biogeographic patterns (e.g., Boyer et al. 2007, Thomas and Hedin 2008, Giribet et al. 2011, Schönhofer et al. 2015, Boyer et al. 2015). The Travunioidea have a temperate Laurasian distribution with species found in eastern Asia, eastern and western North America, and central Europe, with notable absences from central Asia (Figure 2). Recent divergence dating analyses show an ancient origin of Travunioidea dating to >200 million years (Giribet et al. 2010, Sharma and Giribet 2011), although these time estimates should be revisited. Several travunioid lineages show trans-continental distributions. For example, members of the Cladonychiidae are distributed in Europe and eastern and western North America, while the Paranonychidae show a Beringian distribution with genera in western North America, Japan and South Korea. Many other harvestmen show similar broad distributions within the Holarctic including the Sironidae, *Caddo*, *Leiobunum*, and many Dyspnoi (Suzuki 1972b, Suzuki et al. 1977, Shultz and Regier 2009, Hedin et al. 2012a, Hedin et al. 2012b, Schönhofer et al. 2013).

The biological characteristics of essentially all travunioids are quite similar (e.g., dispersal-limited, restricted to cryophilic microhabitats in north temperate latitudes). Regional clades are relatively ancient, allowing ample time for the accumulation of species diversity. In addition, the presence of rare, completely blind, troglobitic species in several different geographic areas speaks to the ancient origin of the Travunioidea. It is likely that troglobitic species, especially those in central Europe, have an unknown diversity concealed by troglomorphy. Similarly, many ancient lineages can be found in the moist, coastal forests of the Pacific Northwest of North America (*Briggsus*, *Metanonychus*) that have likely persisted in refugia through climatic cycles. Additionally, all of these taxa consist of species and subspecies that are short-range endemics (Harvey 2002). Congeneric species syntopy is rare, probably because of ecological niche conservatism that prevents resource partitioning. This ecological niche conservatism likely plays an important role in speciation (see model of Wiens 2004). As such, there is a high potential for species discovery, and recent species-level studies focusing on the travunioid genera *Sclerobunus* and *Cryptomaster* have resulted in the description of new species (Derkarabetian and Hedin 2014, Starrett et al. 2016). Our ongoing studies of Travunioidea are continuing this trend: every genus currently being revised (*Briggsus*, *Erebomaster*, *Theromaster*, *Metanonychus*) shows evidence for new species.

## Supplementary Material

XML Treatment for CRYPTOMASTRIDAE

XML Treatment for TRAVUNIIDAE

XML Treatment for CLADONYCHIIDAE

XML Treatment for PARANONYCHIDAE

## Supplementary materials

Supplementary material 1 Data Table. Taxon sample and UCE sequencing results Data type: species data Explanation note: Samples highlighted in red were sequenced for Starrett et al. (2017). This is an open access article distributed under the terms of the Creative Commons Attribution License (CC BY 4.0), which permits unrestricted use, distribution, and reproduction in any medium, provided the original author and source are credited.Shahan Derkarabetian, James Starrett, Nobuo Tsurusaki, Darrell Ubick, Stephanie Castillo Marshal Hedin

Supplementary material 2 Figures Data type: molecular data Explanation note:
**Figure 1.** Outgroup relationships for the 70% dataset. Nodes are fully supported (e.g., 100 bootstrap, 1.0 posterior probability, etc.) unless otherwise indicated. RAxML tree manually rooted according to BEAST topology. Asterisk in ASTRAL tree indicates node not given support values by default.
**Figure 2.** Representative midgut morphology. Only the right half of the midgut is illustrated. Drawings for Triaenonychoidea (*Synthetonychia*), Grassatores (*Discocyrtus*), Cladonychiidae, and Paranonychidae redrawn from Dumitrescu (1975, 1976). Abbreviations: D1 = diverticulum 1; OD2 = opisthosomal diverticula 2; OD3 = opisthosomal diverticula 3.
**Figure 3.** Representative genital morphology and distribution of *Peltonychia* in Europe. Records from Slovenia and Trieste, Italy (red) are in error. Genitalia from left to right: *P. clavigera*, redrawn from Thaler (1996); *P. leprieurii*, redrawn from Chemini (1985); *P. gabria*, redrawn from Martens (1978).
**Figure 4.** SEM penis morphology of *Yuria* and Paranonychidae. Scale bars 500 mm. This is an open access article distributed under the terms of the Creative Commons Attribution License (CC BY 4.0), which permits unrestricted use, distribution, and reproduction in any medium, provided the original author and source are credited.Shahan Derkarabetian, James Starrett, Nobuo Tsurusaki, Darrell Ubick, Stephanie Castillo Marshal Hedin
