# Supplementary material for: A stable phylogenomic classification of Travunioidea (Arachnida, Opiliones, Laniatores) based on sequence capture of ultraconserved elements
Source: Zookeys. 2018 May 28;(760):1–36. doi: 10.3897/zookeys.760.24937 (PMC5986891; doi:10.3897/zookeys.760.24937)
Supplement: Supplementary material 2 — Figures [file zookeys-760-001-s002.pdf]

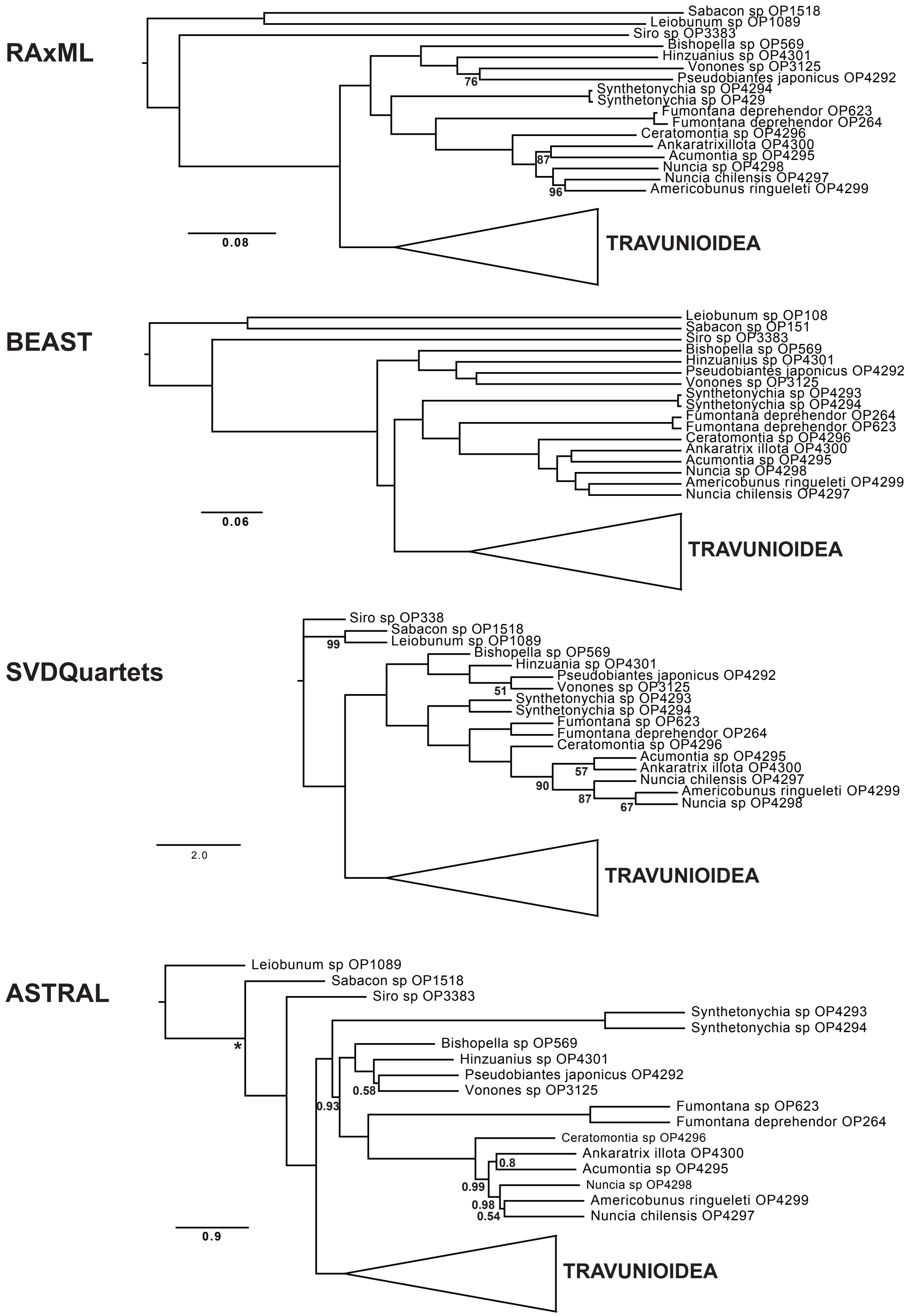

Figure 1. Outgroup relationships for the 70% dataset. Nodes are fully supported (e.g., 100 bootstrap, 1.0 posterior probability, etc.) unless otherwise indicated. RAxML tree manually rooted according to BEAST topology. Asterisk in ASTRAL tree indicates node not given support values by default.

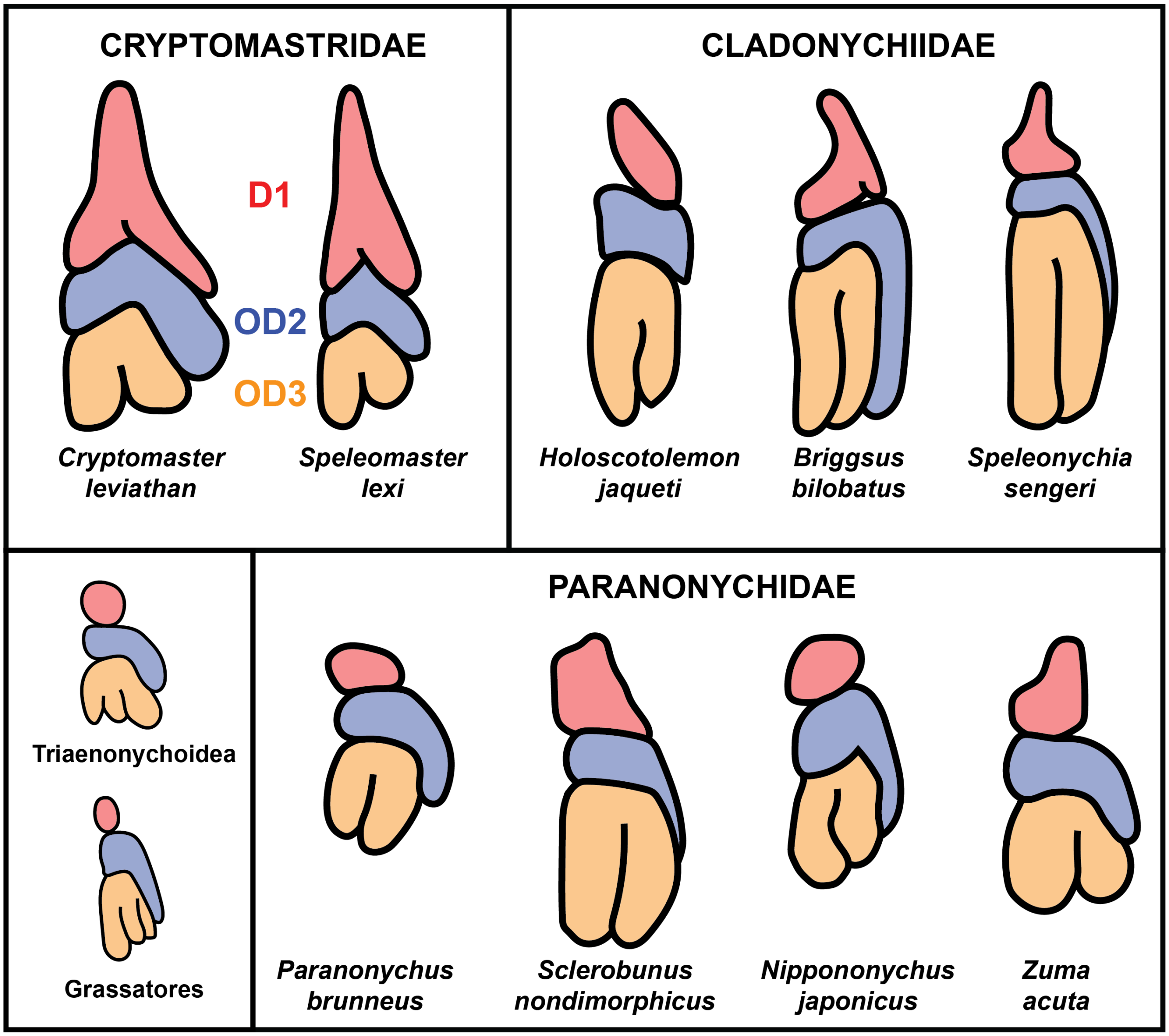

Figure 2. Representative midgut morphology. Only the right half of the midgut is illustrated. Drawings for Triaenonychoidea (*Synthetonychia*), Grassatores (*Discocyrtus*), Cladonychiidae, and Paranonychidae redrawn from Dumitrescu (1975, 1976). Abbreviations: D1 = diverticulum 1; OD2 = opisthosomal diverticula 2; OD3 = opisthosomal diverticula 3.

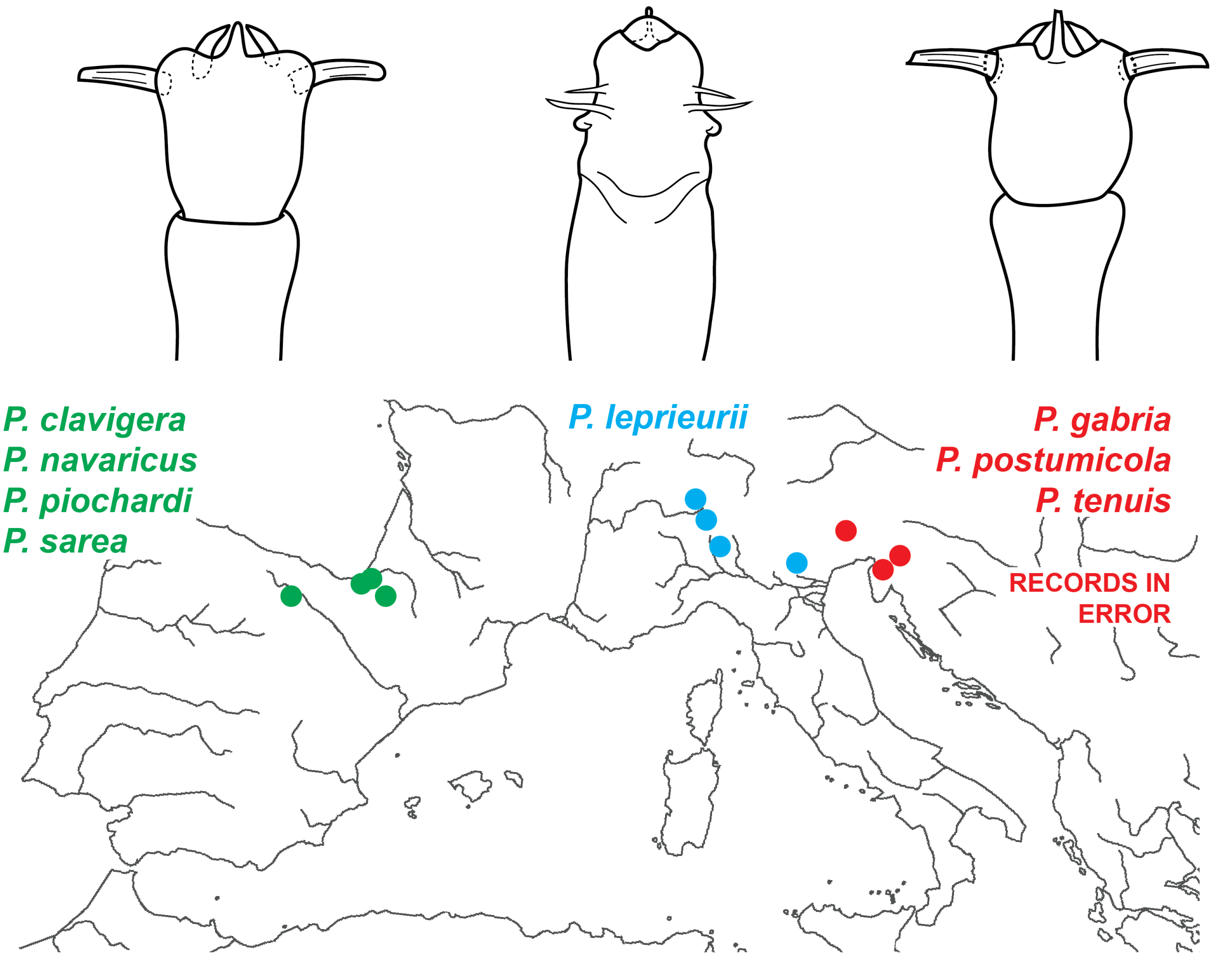

Figure 3. Representative genital morphology and distribution of *Peltonychia* in Europe. Records from Slovenia and Trieste, Italy (red) are in error. Genitalia from left to right: *P. clavigera*, redrawn from Thaler (1996); *P. leprieurii*, redrawn from Chemini (1985); *P. gabria*, redrawn from Martens (1978).

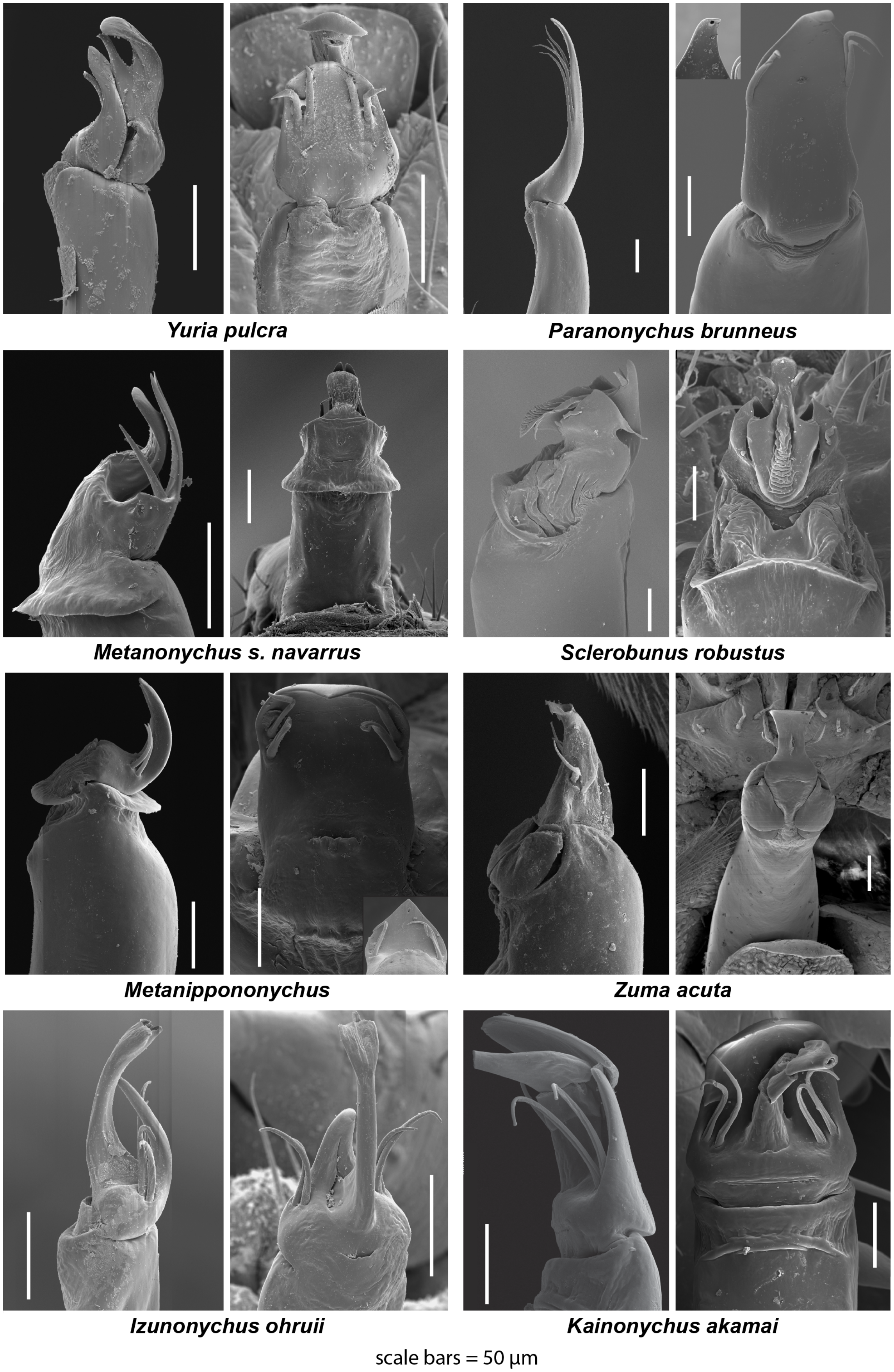

Figure 4. SEM penis morphology of *Yuria* and Paranonychidae. Scale bars = 500 µm.
